# Supplementary material for: Vaccinating to Protect Others: The Role of Self-Persuasion and Empathy among Young Adults
Source: Vaccines (Basel). 2022 Apr 2;10(4):553. doi: 10.3390/vaccines10040553 (PMC9029351; doi:10.3390/vaccines10040553)
Supplement: Supplementary file 1 [file vaccines-10-00553-s001.zip › vaccines-1643916-supplementary.pdf]

Supplementary Materials to: Vaccinating to protect others. The role of self-persuasion and empathy among young adults

Supplementary File 1. *Polish and English versions of the questionnaires*

1. ***Utility beliefs***

English

Getting vaccinated against COVID-19 seems to me, to be:

1. very stupid 1 2 3 4 5 6 7 very wise
2. unprotective 1 2 3 4 5 6 7 protective
3. very harmful 1 2 3 4 5 6 7 very beneficial

Polish

Zaszczepienie się na COVID-19 wydaje mi się:

1. bardzo głupie 1 2 3 4 5 6 7 bardzo rozsądne
2. nieochraniające 1 2 3 4 5 6 7 bardzo ochraniające
3. bardzo szkodliwe 1 2 3 4 5 6 7 bardzo korzystne

2. ***Control beliefs***

English

1. If I wanted to, I could go to a medical facility with no problems whatsoever and get vaccinated, when that becomes possible.

completely disagree 1 2 3 4 5 6 7 completely agree

2. It's mostly up to me, whether I get vaccinated against COVID-19 when a vaccine becomes available.

completely disagree 1 2 3 4 5 6 7 completely agree

3. Getting vaccinated against COVID-19 would be for me:

very hard 1 2 3 4 5 6 7 very easy

4. How much control will you have over getting vaccinated against COVID-19?

no control at all 1 2 3 4 5 6 7 full control

Polish

1. Gdybym chciał/-a mógłbym/mogłabym bez najmniejszego problemu udać się do placówki medycznej i zaszczepić się przeciw COVID-19, kiedy będzie to już możliwe.

zupełnie się nie zgadzam 1 2 3 4 5 6 7 całkowicie się zgadzam

2. To przede wszystkim do mnie należy decyzja, czy zaszczepię się przeciw COVID-19, kiedy będzie to już możliwe.

zupełnie się nie zgadzam 1 2 3 4 5 6 7 całkowicie się zgadzam

3. Zaszczepienie się przeciw COVID-19, kiedy będzie to już możliwe, byłoby dla mnie:

bardzo trudne 1 2 3 4 5 6 7 bardzo łatwe

4. Jaką kontrolę będziesz miał/a nad zaszczepieniem się na COVID-19?
brak kontroli 1 2 3 4 5 6 7 pełna kontrola

3. *Social norm beliefs*

English

1. Most of the people, who are of importance to me, would praise me for self-vaccinating against COVID-19.
2. Most of the people important to me would probably think I should get vaccinated against COVID-19.
3. People whose opinion I care about would say that it is a good idea for me to get vaccinated against COVID-19.

Polish

1) Większość osób, które są dla mnie ważne, pochwaliłaby mnie za zaszczepienie się na COVID-19.
2) Większość ważnych dla mnie ludzi zapewne uznałaby, że powinienem/powinnam zaszczepić się na COVID-19.
3) Ludzie, na których opinii mi zależy, stwierdziliby, że to dobry pomysł, abym za zaszczepił się na COVID-19.

4. *Moral norm beliefs*

English

1. I feel personally accountable for reducing the risk of infecting others during the COVID-19 pandemic by self-vaccinating.
2. Getting vaccinated against COVID-19 should be a moral obligation for all people.
3. Responsible behavior toward others, especially the sick and elderly, requires that I get vaccinated against COVID-19 when that becomes possible.

Polish

1) Czuję się osobiście odpowiedzialny/a za ograniczenie ryzyka zakażenia innych osób poprzez za zaszczepienie się na COVID-19, kiedy będzie to już możliwe.
2) Zaszczepienie się na COVID-19 powinno być moralnym obowiązkiem wszystkich ludzi.
3) Odpowiedzialne zachowanie wobec innych osób, zwłaszcza osób chorych i starszych, wymaga abym zaszczepił się na COVID-19, kiedy będzie to już możliwe

5. *Vaccination intention*

English

When the COVID-19 vaccine becomes available I intend to:

1. Search for information as of where, when and how I can get vaccinated against COVID-19.
2. Make an appointment at a medical facility in order to get vaccinated against COVID-19.
3. Vaccinate myself against COVID-19.

Polish

Gdy będzie dostępna szczepionka na koronawirusa zamierzam:

1. Poszukać informacji na temat tego, gdzie, kiedy i w jaki sposób mogę zaszczepić się przeciw COVID-19
2. Umówić się na wizytę w placówce medycznej w celu zaszczepienia się przeciw COVID-19.
3. Zaszczepić się przeciw COVID-19.

Supplementary File 2 *Experimental manipulation and control in Study 1 in Polish and English*

a. Direct persuasion

Polish

Głównym celem badania jest opracowanie listy przekonujących argumentów na rzecz zaszczepienia się przeciw COVID-19, które mogą być zastosowane w przyszłości do zachęcania ludzi do szczepienia się, kiedy będzie już dostępna na rynku darmowa i skuteczna szczepionka.

Państwa oceny zostaną wykorzystane w celu wyselekcjonowania najbardziej przekonującego argumentu.

Bardzo prosimy o uważne zapoznanie się dwoma argumentami stworzonymi przez Światową Organizacją Zdrowia (WHO) zachęcającymi do zaszczepiania się przeciw COVID-19, kiedy będzie już dostępna na rynku darmowa i skuteczna szczepionka. Prosimy o ocenę, w jakim stopniu zgadzasz się z podanymi argumentami, posługując się następującą skalą, gdzie:
1 – zupełnie się nie zgadzam
2 – nie zgadzam się
3 – raczej nie zgadzam się
4 – ani się zgadzam ani się nie zgadzam
5 – raczej zgadzam się
6 – zgadzam się
7 – całkowicie się zgadzam

1. Chorując bezobjawowo na COVID-19, nie wiemy, że jesteśmy chorzy, ale możemy zarażać inne osoby. Dlatego szczepiąc się chronimy zdrowie starszych członków naszej rodziny (np. babci, dziadka), których widujemy.
2. Szczepienie się chroni przed zachorowaniem na COVID-19. Nawet osoby młode, lekko przechodząc zachorowanie na COVID-19, są narażone na poważne powikłania, jak niewydolność serca.

English

The main goal of this study is to develop a list of compelling arguments in favour of getting vaccinated against COVID-19. The arguments might be used in the future to encourage people to get vaccinated when a free and effective vaccine is available.

Your evaluations will be used to select the most persuasive argument.

Please read carefully the two arguments created by the World Health Organization (WHO) encouraging vaccination against COVID-19 when a free and effective vaccine becomes available. Please rate the extent to which you agree with those arguments using the following scale, where:
1 - I completely disagree
2 - I do not agree
3 - I rather disagree
4 - I neither agree nor disagree
5 - I rather agree
6 - I agree
7 - I completely agree

1. By becoming asymptomatically sick for COVID-19, we do not know whether we are sick, but we can infect others. Therefore, by getting vaccinated, we protect the health of older members of our family (e.g., grandmother, grandfather), with whom we meet.
2. Getting vaccinated protects against contracting COVID-19. Even young people who usually are only mildly affected by COVID-19 are at risk for serious complications, such as heart failure.

b. Experimental group: self

Polish
Głównym celem badania jest opracowanie listy przekonujących argumentów na rzecz zaszczepienia się przeciw COVID-19, które mogą być zastosowane w przyszłości do zachęcania ludzi do szczepienia się, kiedy będzie już dostępna na rynku darmowa i skuteczna szczepionka.

Bardzo prosimy o samodzielne wymyślenie i napisanie co najmniej dwóch mocnych argumentów na rzecz zaszczepiania się przeciw COVID-19, kiedy będzie już dostępna na rynku darmowa i skuteczna szczepionka. Argumenty powinny dotyczyć wyłącznie ochrony zdrowia osoby szczepiącej się (a nie jej otoczenia społecznego).

Przykładowy argument:
Szczepienie się chroni przed zachorowaniem na COVID-19. Nawet osoby młode, lekko przechodząc zachorowanie na COVID-19, są narażone na poważne powikłania, jak niewydolność serca.

Stworzone przez Państwa argumenty zostaną ocenione przez przeszkolone osoby w celu wyselekcjonowania najbardziej przekonujących argumentów.

English
The main goal of this study is to develop a list of compelling arguments in favour of getting vaccinated against COVID-19. The arguments might be used in the future to encourage people to get vaccinated when a free and effective vaccine is available.

Please think independently of at least two strong arguments in favour of getting vaccinated against COVID-19 when a free and effective vaccine is available, and write them down. The arguments should only concern protecting the health of the person getting vaccinated (not their social environment).

Example:

Getting vaccinated protects against contracting COVID-19. Even young people who usually are only mildly affected by COVID-19 are at risk for serious complications, such as heart failure.

Your arguments will be evaluated by trained individuals to select the most persuasive arguments.

c. Experimental group: others

Polish
Głównym celem badania jest opracowanie listy przekonujących argumentów na rzecz zaszczepienia się przeciw COVID-19, które mogą być zastosowane w przyszłości do zachęcania ludzi do szczepienia się, kiedy będzie już dostępna na rynku darmowa i skuteczna szczepionka.

Bardzo prosimy o samodzielne wymyślenie i napisanie co najmniej dwóch mocnych argumentów na rzecz zaszczepiania się przeciw COVID-19, kiedy będzie już dostępna na rynku darmowa i skuteczna szczepionka. Argumenty powinny dotyczyć wyłącznie ochrony zdrowia innych osób, niż osoba szczepiąca się (nie jej zdrowia), np. tych które nie będą mogły zaszczepić się (osoby starsze, chore, niemowlęta).

Przykładowy argument:
Chorując bezobjawowo na COVID-19, nie wiemy, że jesteśmy chorzy, ale możemy zarażać inne osoby. Dlatego szczepiąc się, chronimy zdrowie starszych członków naszej rodziny (np. babci, dziadka), których widujemy.

Stworzone przez Państwa argumenty zostaną ocenione przez przeszkolone osoby w celu wyselekcjonowania najbardziej przekonujących argumentów.

English
The main goal of this study is to develop a list of compelling arguments in favour of getting vaccinated against COVID-19. The arguments might be used in the future to encourage people to get vaccinated when a free and effective vaccine is available.

Please think independently of at least two strong arguments in favour of getting vaccinated against COVID-19 when a free and effective vaccine is available, and write them down. The arguments should only concern protecting the health of people other than the one getting the vaccine. For instance those who cannot be vaccinated (old people, sick people, infants)

Example:
By becoming asymptomatically sick for COVID-19, we do not know whether we are sick, but we can infect others. Therefore, by getting vaccinated, we protect the health of older members of our family (e.g., grandmother, grandfather), with whom we meet.

Your arguments will be evaluated by trained individuals to select the most persuasive arguments.

Supplementary File 3 *Experimental manipulation in Study 2 in Polish and English*

A. **Instructions:**
   a. **Polish**

Ocena grafik informujących o szczepieniach przeciw koronawirusowi

Głównym celem badania jest ocena grafik informacyjnych związanych z możliwością zaszczepienia się przeciw COVID-19 (koronawirusowi), które mogą być zastosowane w przyszłości, kiedy zostanie stworzona skuteczna i darmowa szczepionka przeciw koronawirusowi. Prosimy, wybierz jedną z dwóch poniżej prezentowanych grafik, która - według Ciebie - jest bardziej estetyczna i dopasowana do treści komunikatu.

[GRAFIKA; możliwość wyboru preferowanej grafiki]

Uwarunkowania gotowości do zaszczepienia się przeciw koronawirusowi
Celem drugiej części tego badania jest określenie czynników psychologicznych wpływających na gotowość do zaszczepienia się przeciw koronawirusowi (COVID-19), kiedy zostanie opracowana skuteczna i darmowa szczepionka.

b. **English**

Evaluation of coronavirus vaccination infographics
The primary purpose of this research is to evaluate infographics related to the COVID-19 (coronavirus) vaccination. Those infographics will be used in the future when an effective and free coronavirus vaccine is developed. Please select one of the two graphics below that you feel is more aesthetically pleasing and best matches the comment on the graphic.

[IMAGE; choice of the preferred graphic]

Determinants of willingness to vaccinate against coronavirus
The purpose of the second part of this study is to determine the psychological factors influencing willingness to be vaccinated against coronavirus (COVID-19) when an effective and free vaccine is developed.

B. Graphics:

a. Direct persuasion

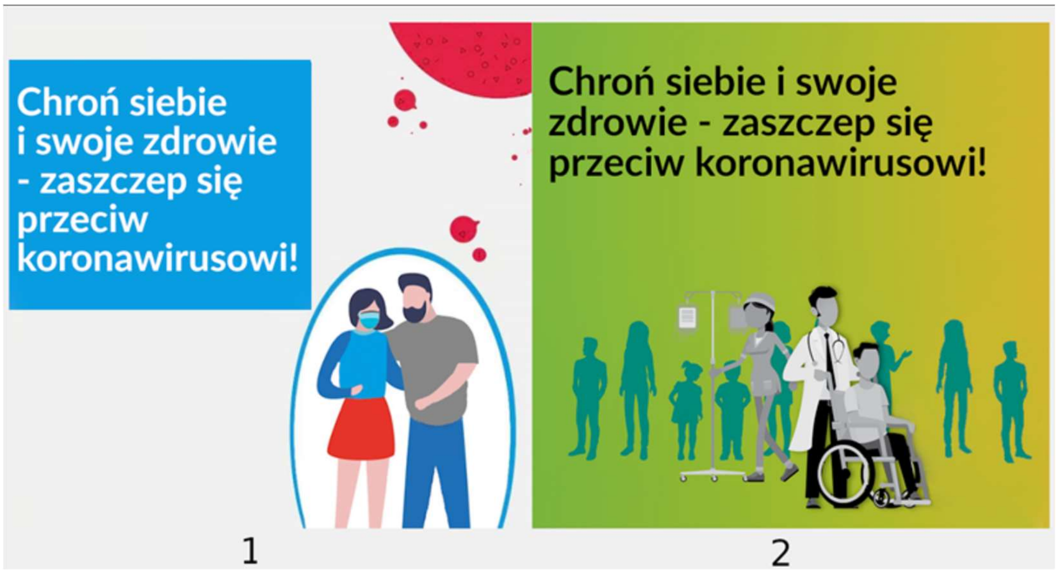

English: Protect yourself and your own health - take the coronavirus vaccine!

b. Manipulation focused on health of others

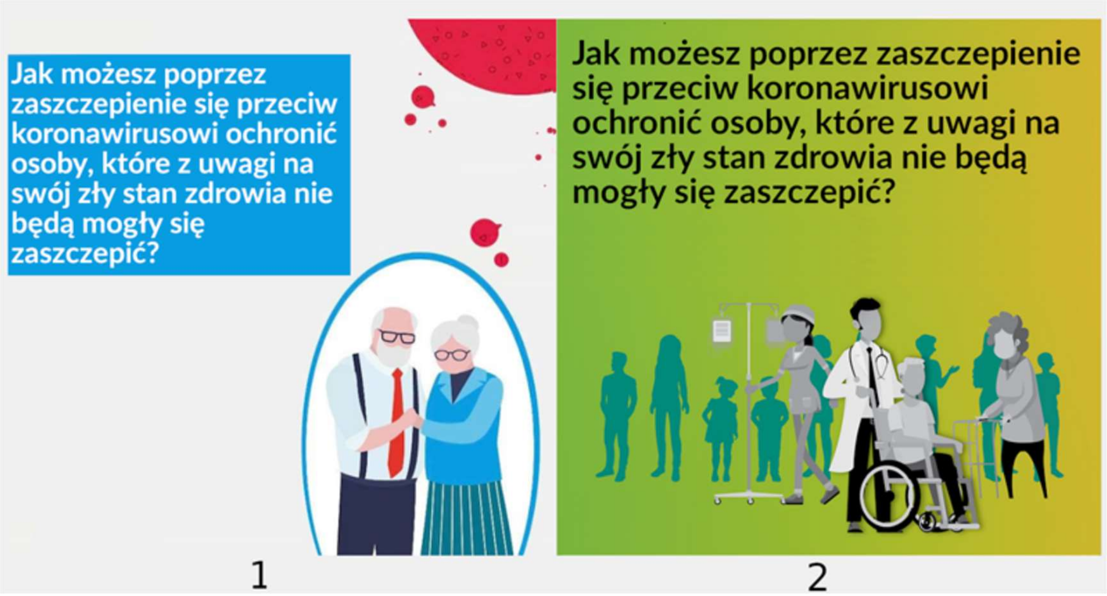

English: How can you protect other people, especially those who cannot take the vaccine due to health problems, through vaccinating?

Supplementary File 4 *Tables*

Supplementary Table 1

Descriptive statistics for each group in Study 1

Measure	Experimental group: others		Experimental group: self		Direct persuasion	
	M	*SD*	*M*	*SD*	*M*	*SD*
1. Empathy	3.48	.70	3.34	.65	3.36	.69
2. Intention	5.51	1.75	4.99	2.18	5.01	1.93
3. Utility beliefs	5.76	1.18	5.12	1.82	5.16	1.67
4. Control beliefs	5.28	1.38	5.16	1.65	5.06	1.51
5. Social norm beliefs	5.06	1.74	4.72	1.92	4.48	1.81
6. Moral norm beliefs	5.42	1.60	4.76	2.11	4.76	1.92
7. Sex	.24	.44	.20	.46	.20	.42
8. K-COVID-19	.80	.72	.70	.72	.53	.67

Supplementary Table 2

Means, standard deviations and factor loading for all items in Study 1

Factor	Item	Mean	SD	Factor Loading		
				CFA	Initial Model	Final Model
Empathy	EM1	3.94	.96	.502	-	-
	EM2	4.10	.83	.229	-	-
	EM3	3.60	1.22	.514	.705	.706
	EM4	3.97	.83	.212	-	-
	EM5	3.45	1.11	.572	.606	.606
	EM6	3.96	1.00	.564	.365	.365
	EM7	3.88	1.16	.570	.406	.406

	EM8	3.53	1.20	.511	.660	.659
	EM9	4.13	.94	.308	-	-
	EM10	3.83	1.02	.651	.441	.441
	EM11	1.76	.79	.123	-	-
	EM12	2.54	1.03	.339	-	-
	EM13	3.20	1.22	.457	.643	.643
	EM14	3.58	1.03	.295	-	-
	EM15	2.07	1.02	.333	-	-
	EM16	4.03	.79	.182	-	-
	EM17	3.06	1.05	.317	.523	.524
	EM18	2.96	1.23	.211	.403	.403
	EM19	3.01	1.12	.244	-	-
	EM20	2.55	1.20	.427	.479	.479
	EM21	3.52	1.43	.665	.582	.582
Intention	I1	5.46	1.93	.875	.871	.871
	I2	5.02	2.04	.976	.977	.977
	I3	5.11	2.08	.987	.990	.990
Utility beliefs	U1	5.62	1.73	.972	.972	.972
	U2	5.26	1.68	.854	.853	.851
	U3	5.25	1.91	.844	.848	.847
Control beliefs	C1	5.25	1.86	.828	.869	.873
	C2	5.70	1.52	.251	-	-
	C3	5.04	1.89	.827	.893	.897
	C4	5.22	1.68	.411	.436	.438
Social norm beliefs	S1	4.77	1.86	.902	.894	.894
	S2	4.69	1.90	.953	.954	.954
	S3	4.83	1.96	.963	.969	.969
Moral norm beliefs	M1	5.05	1.91	.900	-	-
	M2	4.72	2.08	.899	-	-
	M3	5.27	2.00	.846	-	-

Supplementary Table 3

Means, standard deviations and factor loading for all items in Study 2

Factor	Item	Mean	SD	Factor Loading		
				CFA	Initial Model	Final Model
Intention	I1	5.36	1.87	.866	.852	.852
	I2	4.90	2.02	.943	.956	.956
	I3	5.05	2.06	.987	.987	.987
Utility beliefs	U1	5.55	1.78	.970	-	-
	U2	5.20	1.66	.862	-	-
	U3	5.18	1.76	.872	-	-
Control beliefs	C1	5.36	1.75	.719	.767	.767
	C2	5.83	1.40	.293	-	-
	C3	5.14	1.66	.834	.920	.922
	C4	5.53	1.48	.216	-	-
Social norm beliefs	S1	4.84	1.83	.906	.903	.903
	S2	4.80	1.86	.900	.911	.911
	S3	4.95	1.90	.987	.981	.981
	M1	5.12	1.88	.922	-	-

Moral norm beliefs	M2	4.67	2.06	.919	-	-
	M3	5.19	1.97	.956	-	-

Supplementary Table 4

Descriptive statistics for each group in Study 2

**Measure**	**Self-persuasion**		**Direct persuasion**	
	M	*SD*	*M*	*SD*
1. Intention	5.23	1.87	4.99	1.92
2. Utility beliefs	5.38	1.57	5.23	1.68
3. Control beliefs	5.26	1.64	5.26	1.49
4. Social norm beliefs	4.91	1.75	4.82	1.80
5. Moral norm beliefs	4.95	1.90	5.04	1.86
6. Sex	.27	.45	.34	.51
7. K-COVID-19	.70	.67	.66	.66

Supplementary Table 5

Direct effects, indirect effects and total effects in Study 1 and Study 2

N	Variables	Direct effect	Indirect effect	Total effect
	**Study 1**			
1	U → I	.458***	-	.458***
2	S → I	.246**	-	.246**
3	C → I	.271**	-	.271**
4	Se → U	-.008	-	-.008
5	Se → S	.061	-	.061
6	Se → C	.050	-	.050
7	Se → I	-.034	-	-.009
	Se → U → I	-	-.004	-
	Se → S → I	-	.015	-
	Se → C → I	-	.014	-
8	O → U	.176**	-	.176**
9	O → S	.154*	-	.154*
10	O → C	.070	-	.070
11	O → I	-.029	-	.108
	O → U → I	-	.081*	-
	O → S → I	-	.038†	-
	O → C → I	-	.019	-
12	E → U	.176*	-	.176*
13	E → S	.156*	-	.156*
14	E → C	.245**	-	.245**
15	E → I	.012	-	197**
	E → U → I	-	.080*	-
	E → S → I	-	.038	-
	E → C → I	-	.066*	-
16	Sx → E	-.324***	-	-.324***
17	Sx → U	.116	-	.059
	Sx → E → U	-	-.057†	-
18	Sx → S	.091	-	.041

	Sx → E → S	-	-.050	-
19	Sx → C	.081	-	.002
	Sx → E → C	-	-.079*	-
20	Sx → I	.001	-	.94
	Sx → E → I	-	-.004	-
	Sx → U → I	-	.053	-
	Sx → E → U → I	-	-.026	-
	Sx → S → I	-	.022	-
	Sx → E → S → I	-	-.012	-
	Sx → C → I	-	.022	-
	Sx → E → C → I	-	-.021	-
	**Study 2**			
1	S → I	.394***	-	.394***
2	C → I	.543***	-	.543***
3	G → S	-.009	-	-.009
4	G → C	-.044	-	-.044
5	G → I	.064*	-	.036
	G → S → I	-	-.004	-
	G → C → I	-	-.024	-
6	Sx → S	.088	-	.088
7	Sx → C	.089	-	.089
8	Sx → I	-.072*	-	.011
	Sx → S → I	.-	.035	-
	Sx → C → I	-	.049	-

Note. *$p < .05$, ** $p < .01$, *** $p < .001$; † = 0.51/0.52; Sex was dummy coded as 0 = Female, 1 = Male
U - Utility beliefs, C - Control Beliefs, S - Social norm beliefs, Se - Experimental manipulation: self, O - Experimental manipulation: others, E - Empathy, I - Vaccination Intention, Sx - Sex, G - Group
